# Analysis of Geometric Shifts and Proper Setup-Margin in Prostate Cancer Patients Treated With Pelvic Intensity-Modulated Radiotherapy Using Endorectal Ballooning and Daily Enema for Prostate Immobilization

**DOI:** 10.1097/MD.0000000000002387

**Published:** 2016-01-15

**Authors:** Songmi Jeong, Jong Hoon Lee, Mi Joo Chung, Sea Won Lee, Jeong Won Lee, Dae Gyu Kang, Sung Hwan Kim

**Affiliations:** From the Department of Radiation Oncology, St. Vincent Hospital, College of Medicine, The Catholic University of Korea, Suwon (SJ, JHL, MJC, SWL, DGK, SHK); and Department of Radiation Oncology, Kyungpook National University Hospital, Daegu, Korea (JWL).

## Abstract

We evaluate geometric shifts of daily setup for evaluating the appropriateness of treatment and determining proper margins for the planning target volume (PTV) in prostate cancer patients.

We analyzed 1200 sets of pretreatment megavoltage-CT scans that were acquired from 40 patients with intermediate to high-risk prostate cancer. They received whole pelvic intensity-modulated radiotherapy (IMRT). They underwent daily endorectal ballooning and enema to limit intrapelvic organ movement. The mean and standard deviation (SD) of daily translational shifts in right-to-left (X), anterior-to-posterior (Y), and superior-to-inferior (Z) were evaluated for systemic and random error.

The mean ± SD of systemic error (Σ) in X, Y, Z, and roll was 2.21 ± 3.42 mm, −0.67 ± 2.27 mm, 1.05 ± 2.87 mm, and −0.43 ± 0.89°, respectively. The mean ± SD of random error (δ) was 1.95 ± 1.60 mm in X, 1.02 ± 0.50 mm in Y, 1.01 ± 0.48 mm in Z, and 0.37 ± 0.15° in roll. The calculated proper PTV margins that cover >95% of the target on average were 8.20 (X), 5.25 (Y), and 6.45 (Z) mm. Mean systemic geometrical shifts of IMRT were not statistically different in all transitional and three-dimensional shifts from early to late weeks. There was no grade 3 or higher gastrointestinal or genitourianry toxicity.

The whole pelvic IMRT technique is a feasible and effective modality that limits intrapelvic organ motion and reduces setup uncertainties. Proper margins for the PTV can be determined by using geometric shifts data.

## INTRODUCTION

Prostate cancer is cured by multimodality treatment and intermediate to high-risk prostate cancer is mainly treated with radiotherapy.^1^ The proper extent of the radiation field, whether prostate only radiotherapy (PORT) or whole pelvic radiotherapy (WPRT), is still somewhat controversial. The probability of lymph node metastasis is high in some patient groups.^2^ Thus, the effectiveness of elective pelvic irradiation is supported by randomized and retrospective series.^3,4^ These studies showed that WPRT achieved better outcomes in disease control, as compared to PORT in intermediate to high-risk group prostate cancer patients.

Image-guided radiotherapy (IGRT) and intensity-modulated radiotherapy (IMRT) are accepted as efficient radiation techniques for prostate cancer treatment.^5^ These techniques facilitate the delivery of higher dose to the planning target volume (PTV) in conjunction with lower dose to the normal tissue than conventional radiotherapy. The simultaneous integrated boost (SIB) technique delivers even better conformal radiation to gross tumor and pelvic lymphatics.^6^ Clinical outcomes such as biochemical relapse-free survival are improved and incidence of toxicities of WPRT are similar or decreased with these techniques, as compared to conventional radiotherapy.^7^ The accuracy of daily setup and organ movement are important issues in whole pelvic SIB-IMRT because not only prostate irradiation but also elective nodal irradiation is an important aspect of treatment accuracy. It is necessary to minimize intrapelvic organ movements in daily radiation procedures to the highest extent possible by limiting movements of prostate, rectum, and bladder by applying organ localization procedures. For example, bladder emptying, rectal enema, and endorectal balloon insertion are good ways to limit organ movement.^8^ The relative position of the prostate in pelvic bony anatomy is useful for the daily setup verification, and the degree of patient setup shifts is thought to be the major determinant for the proper PTV margin in radiotherapy.

We treated intermediate to high-risk prostate cancer patients in our institution by using whole pelvic SIB-IMRT and IGRT. We limited intrapelvic organ movements by using daily bladder emptying, rectal enema, and endorectal ballooning. Megavoltage-CT scans were acquired before each treatment to obtain data for daily geometric shifts. In this study, we analyzed geometric shifts of daily patient setups to evaluate the appropriateness of our treatments and to calculate proper PTV margins.

## MATERIALS AND METHODS

### Patients

Prostate cancer patients who received radiotherapy with Helical TomoTherapy in our institution from 2011 to 2014 were evaluated. Clinical staging work-up included digital rectal examination, complete blood count, liver and renal function test, level of prostate-specific antigen (PSA), chest and abdomen CT, and pelvic MRI before radiotherapy. Bone scan was done in all patients. All patients had histologically proven adenocarcionoma of the prostate and were diagnosed as cT2–3 according to the American Joint Committee on Cancer Staging System, 7th edition. They were intermediate to high-risk group prostate cancer patients according to the National Cancer Center Network Guideline. Patients who had irradiated prostate only were excluded from the study, and 40 patients who received radiation to both prostate and pelvic lymphatics using the SIB-IMRT technique of TomoTherapy were analyzed. Institutional review board approval was obtained before collecting the patient data (VC15RISI0016).

### Simulation and Planning

**FIGURE 1:**
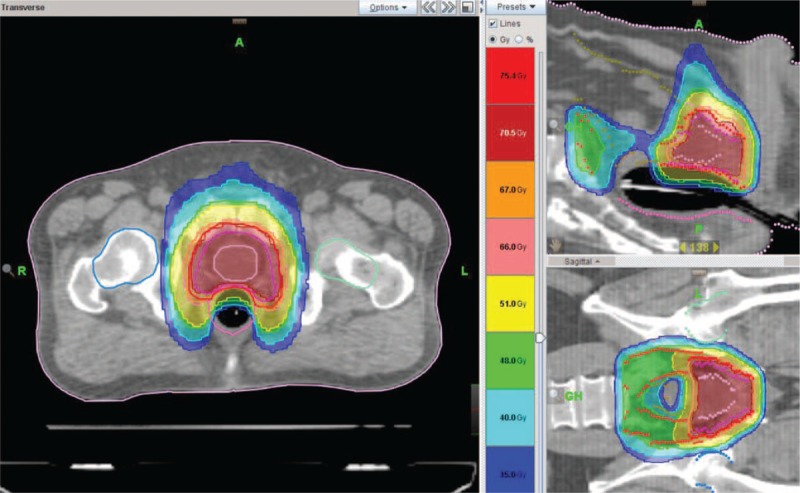
A prostate cancer patient received pelvic irradiation with TomoTherapy using a simultaneous integrated boost technique.

For radiation simulation, CT scan was performed at 3-mm slice thickness. Vacuumed lock cushion covering the entire body was used for immobilizing the patient in the supine position. Bladder emptying, rectal enema, and endorectal balloon insertion were done for simulation as well as for each treatment to minimize intrapelvic organ movements. Endorectal balloon was inserted and inflated with the same volume of 60 cc air. There were markings at the end of the balloon for indicating the location of anal verge in each patient. The T2-weighted MR image was also obtained in the same position as endorectal ballooning insertion. MR images were fused to simulation CT images for proper target contouring. For the SIB-IMRT plan, the raw dosimetric data set of each patient was transferred to the TomoTherapy Hi-Art version 4.0 planning system (Accuray, Sunnyvale, CA) (Figure 1).

The gross target volume (GTV) was the prostate and metastatic lymph node. The clinical target volume (CTV) included GTV and pelvic lymphatics. The CTV for pelvic lymphatics was defined from the level of the common iliac bifurcation to the obturator lymphatics above the level of symphysis pubis. The planning target volume (PTV) was from 7 to 10 mm expansion anterior, superior, and inferior from the CTV, and posterior expansion from the CTV while excluding the involved rectum. The prescribed radiation dose of the PTV of prostate and lymphatics was 63 to 70.5 Gy and 48 to 54 Gy in 30 fractions, respectively. To reduce the setup uncertainty of each patient, daily megavoltage CT (MVCT) images were obtained before irradiation. Obtained MVCT images were fused with the planning CT images and were adjusted for proper setup verification and treatment. Because all radiotherapy was intended to simultaneously target the prostate and pelvic lymphatics, initial autoregistration of daily MVCT to initial planning CT was performed in bone-matched session and followed by manual registration. Radiation oncologists reviewed and checked the fused CT images in each treatment fraction.

### Evaluation of Proper Target Margin

Data for daily geometric shifts of the patient setup in right-to-left (X), anterior-to-posterior (Y), superior-to-inferior (Z), and angle of collimator (roll) were collected. Movement to right, anterior, and superior direction was set as a positive shift, and movement to left, posterior, and inferior direction was set as a negative shift. Geometrical shifts data were used to calculate the proper PTV margin. Appropriateness of daily treatment was by mean and standard deviation (SD) of systemic error (∑) and random error (σ) in the overall patient population. In this study, the systemic error was defined as the shift in geometry between fractionated treatment and the simulation isocenter. The random error was defined as the shift that occurred between consecutive fractions. The proper PTV margin that ensures a minimum average 95% dose to the target was calculated using the equation, ‘margin = 2Σ+0.7δ’.^9^

### Clinical Outcome and Follow-Up

The follow-up period was defined as the date of start of radiation to the date of expiration or the time of analysis, October 2014. Biochemical relapse was defined as the date of first PSA level increase > 2 ng/mL above the PSA nadir after RT, according to the Phoenix definition. PSA assessments were done at 1 and 3 months after radiotherapy, every 3 months for the next 3 years, and every 6 months thereafter. Treatment-related acute and late toxicities were evaluated by physicians using Common Terminology Criteria for Adverse Events (CTCAE) ver. 4.0.

### Statistical Analysis

The tendency of geometric shifts with time factor during the radiation course from early to late week was analyzed by repeated measure analysis of variance. Results of *P* value < 0.05 were considered statistically significant. The correlation between setup uncertainty and several clinical factors including patient age and weight were analyzed by the Student's *t* test.

## RESULTS

The patient characteristics were described in Table 1. The median age of the 40 patients was 71 years (range 52–84 years). The median body weight was 65.8 kg (range 45.5–89 kg). Of the 40 patients, 33 (82.5%) were in the high-risk group; 24 (60%) had cT3 tumors, 21 (52.5%) had initial PSA of > 20 ng/mL, and 11 (27.5%) had Gleason score ≥ 8. Thirty-one patients received androgen deprivation therapy (ADT) with radiotherapy.

**TABLE 1:**
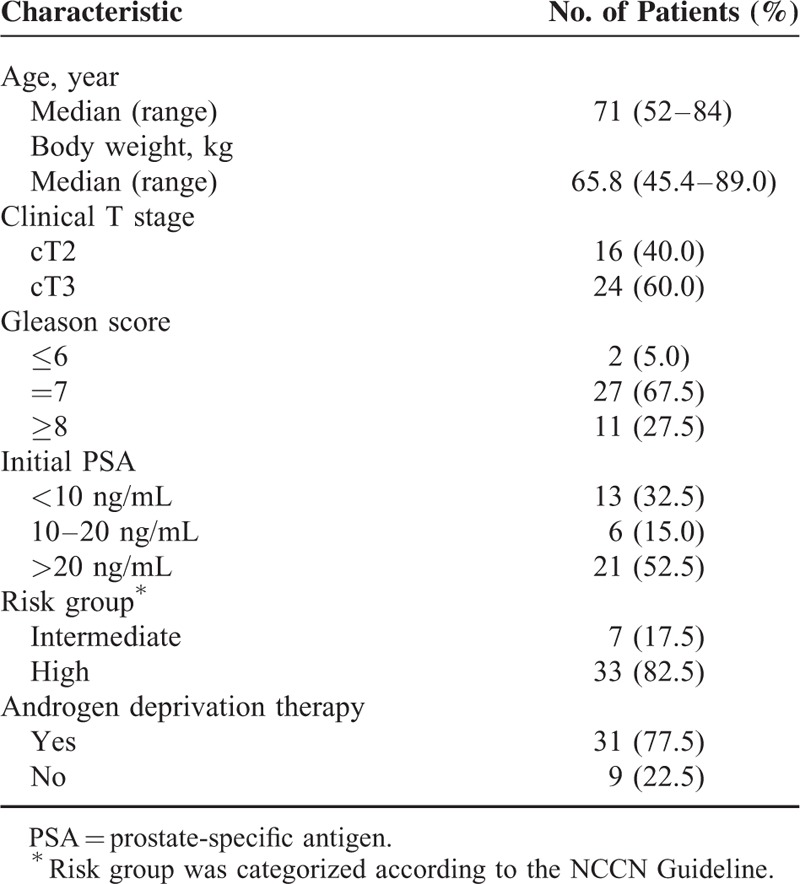
Patient Characteristics (n = 40)

**FIGURE 2:**
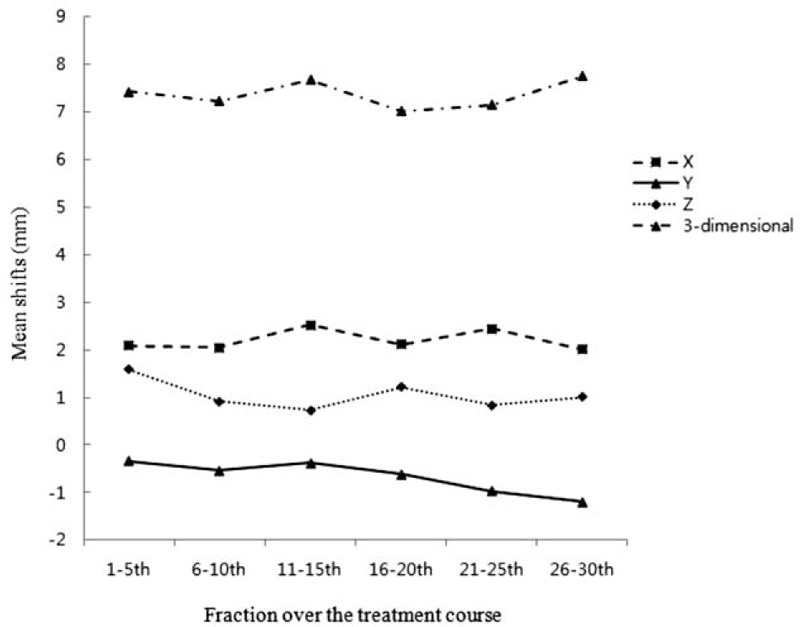
Mean systemic geometrical shifts from early to late weeks.

All patients completed prescribed radiation schedules, and 1200 sets of daily pretreatment MVCT scans were acquired for analyzing the daily geometric shifts. The mean and standard deviation (SD) of systemic geometrical shifts (Σ) in right-to-left (X), anterior-to-posterior (Y), superior-to-inferior (Z) direction, and angle of collimator (roll) were 2.21 ± 3.42 mm, −0.67 ± 2.27 mm, 1.05 ± 2.87 mm, and −0.43 ± 0.89°, respectively. The mean and SD of random geometrical shifts (δ) was 1.95 ± 1.60 mm in X, 1.02 ± 0.50 mm in Y, 1.01 ± 0.48 mm in Z, and 0.37 ± 0.15° in roll. The obtained geometric shifts data and calculated margin were described in Table 2. The calculated proper PTV margins using the analyzed transitional shifts and automated equation, margin = 2Σ + 0.7δ, were 8.20 mm in X, 5.25 mm in Y, and 6.45 mm in Z, respectively. The change of weekly geometric shifts during pelvic irradiation using TomoTherapy was summarized in Table 3. Mean geometrical shifts were not statistically different during the pelvic radiation period in all transitional and three-dimensional shifts from early to late weeks (Figure 2). Clinical factors, patient age, and weight were not correlated with all shifts.

**TABLE 2:**
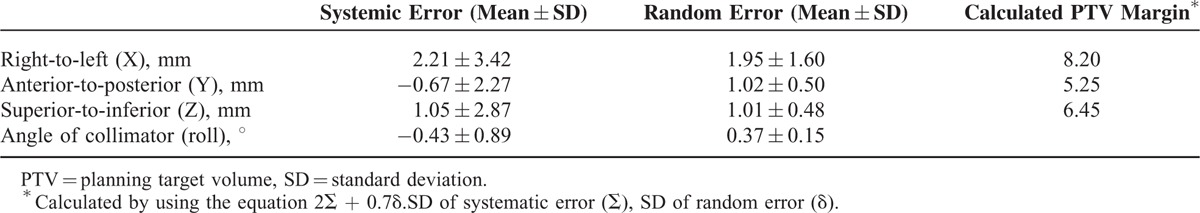
Systemic and Random Geometric Shifts With Calculated Proper Margins

**TABLE 3:**
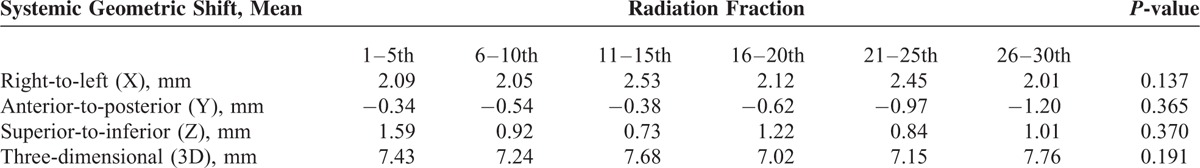
Change of Mean Systemic Geometric Shifts During Radiation Therapy Fractions

The median follow-up time was 16 months (range 2–38 months). All patients reached PSA nadir < 2 ng/mL after radiotherapy. During the follow-up time, only 1 had biochemical recurrence 22 months after the end of radiotherapy and 1 patient expired due to cardiovascular disease after the end of radiotherapy. Acute and chronic toxicities of gastrointestinal and urinary tract were evaluated. Toxicity profiles were described in Table 4. There was no severe acute and late complication > grade 3 during and after treatment and until the follow-up time.

**TABLE 4:**
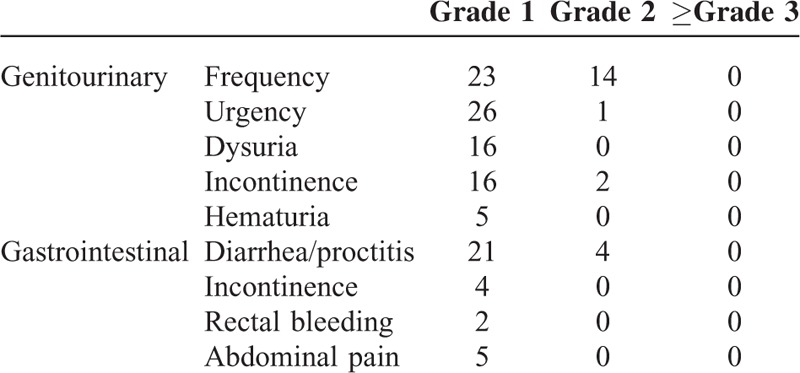
Toxicity Profile of Pelvic Radiotherapy

## DISCUSSION

IMRT is accepted as an effective modality for prostate cancer treatment. Its higher conformality and precise dose delivery allows tight PTV margins. The steep dose gradient between PTV and surrounding at risk organs potentially reduces normal tissue toxicity while delivering higher radiation dose to PTV. Furthermore, prostate cancer has a low α/β ratio of 1.5:2, with higher fraction size that also increases radiobiological effects. However, tight PTV margins increase the risk of geometrical missing if the target position is not well verified at each treatment. Patient body setup and target movement become much more important in IMRT.

Numerous previous PORT studies focused on the movement of the target prostate itself. The prostate position was evaluated by inserting fiducial markers,^10,11^ using the BAT system, and repeated CT scans.^12,13^ However, in the WPRT era, because radiotherapy simultaneously targets prostate and pelvic lymphatics, considering the relative position of prostate and intrapelvic organ to pelvic bony anatomy is more appropriate for more precise and safe treatment than focusing solely on prostate movement. Therefore, well-designed radiotherapy protocol that can effectively control the intrapelvic organ movement is needed in WPRT using IMRT.

Several strategies and protocols were suggested to control variation of intrapelvic organ position. One strategy is daily pelvic CT imaging to verify interfractional rectal or bladder volume changes in each fraction. Among the several prepared RT plans, which differ according to intrapelvic organ and target position changes, it is important to choose the most appropriate plan prior to treatment or adapting new plans over the RT course.^14,15^ However, the strategy of using several RT plans has the potential high risk of increasing total dosimetric uncertainties of treatment.

Another strategy is to limit intrapelvic organ motion itself effectively by applying organ localization procedures as in our protocol, that is, daily emptying bladder with rectal enema and endorectal ballooning. Several studies reported variations of anatomic changes of pelvic organs^16,17^ and Li et al^18^ reported that position changes were mostly due to the dramatic rectal volume change. The use of a rectal balloon is particularly well known for its benefit in prostate immobilization and reduced rectal toxicity.^19,20^ Rectal balloon insertion can effectively control the rectal volume change by balloon inflation with the same air volume at each treatment. Resulting rectal wall distension also reduces high doses to the posterior or lateral rectal wall. Air-filled balloons also have dosimetric benefits of air–tissue interface for reducing the anterior rectal wall dose while maintaining the posterior prostate dose. Teh et al^19^ reported that dose profiles with air cavity is 15% lower and rectal toxicity profiles showed favorable outcome in comparison to the profiles without air cavity.

Well-designed organ immobilization procedures lead to higher patient setup accuracy because geometric shifts that are affected by intrapelvic organ movements might become minimized or negligible. Several published results indicated that mean shifts and SD of systemic error are in the range of 1 to 2 mm and 3 to 8 mm when applying immobilization device and setup-based pelvic bony landmark.^21,22^ Our current results were similar to results of the previous studies. The mean shifts of all patients were 2.21 mm in X, −0.67 mm in Y, 1.05 mm in Z and SD of systemic error were 3.42 mm in X, 2.27 mm in Y, and 2.87 mm in Z, respectively.

Using these geometric shifts data, we calculated proper PTV margins based on the equation, 2Σ+0.7δ, described by Stroom et al.^9^ They suggested the PTV margin based on Gaussian error distributions to deliver at least 95% of the prescribed dose covering 99% of the CTV. Therefore, the calculated proper PTV margins for our radiotherapy protocols are 8.20 mm in X, 5.25 mm in Y, and 6.45 mm in Z. The results suggested that 5 to 10 mm were relatively appropriate PTV margins, as applied in our radiotherapy protocol. Guckenberger et al^23^ likewise treated prostate cancer patients with IMRT-SIB and CT-guided IGRT and also applied 5 to 10 mm CTV-to-PTV margins.

The good clinical outcomes in our study indicated appropriate and effective delivery of radiation treatment. All study patients reached a PSA nadir < 2 ng/mL after radiotherapy. Most patients reached a PSA nadir < 0.2 ng/mL, except for 2 patients whose PSA levels continued to decline until their last follow-up. We could not check additional levels in 1 patient who refused additional testing after PSA reached 0.27 ng/mL at the 14.6 month follow-up, and another patient expired from cardiovascular disease after reaching a 0.21 ng/mL PSA level. Our clinical result was comparable to previous studies, considering that 82.5% of our study patients were in the high-risk group.^5,24,25^ Concomitant with the good outcomes, there was also no severe toxicity > grade 3. The data thus supported that organ movements were well limited with the immobilizing procedures and radiation was actually delivered precisely and effectively as planned.

The factor correlated with geometrical shifts was analyzed in our study. Before the analysis, we assumed that several patient factors, especially patient's body weight might affect the setup accuracy because it is more difficult to immobilize a body with excessive weight. However, in the present study, clinical factors did not show statistically significant differences in geometrical shifts. The time factor, fractions over time from early weeks to late weeks, might affect the degree of geometric shifts because both, the patient and radiation therapist, become experienced with RT procedures over the fractions. However, there were no statistical differences of geometrical shifts over the fractions and changes of weekly mean shifts in all directions were within 1 mm. This result supported the study of Alasti et al^22^ that setup errors occur throughout the radiation course and degree of shifts are unchanged over fractions.

The one possible limitation of this study was that intrafractional change by respiration was uncontrolled. Internal organ motion by respiration is known to generate artifacts in simulation CT images and inaccuracies in contouring. However, the possibility of missing geometrical target is low because we used slow CT in the simulation process. Furthermore, several organ immobilization procedures might effectively reduce not only the degree of intrafractional motion but also the degree of interfractional movement. PET imaging has contributed substantially in oncology by allowing improved clinical staging and guiding appropriate cancer management. Integration with radiotherapy planning via PET-CT simulation could enable improved target delineation in future.^26–28^

The weakest point of our study was the small patient number and relatively short follow-up, as compared to the long overall survival of prostate cancer patients. Small population size may contribute to the statistical insignificance in analysis of correlation of geometric shifts with several factors, and short follow-up may contribute to low biochemical recurrence rates and toxicities. Further studies in future with a larger population size and longer follow-up time are needed to verify the actual outcomes and factors that contribute to the geometric shifts.

Nevertheless, we acquired each patient's entire geometric shifts data during complete RT courses and these over thousand fractions data were used to evaluate proper target margins. Analyses of whole geometric shifts data itself is valuable for quality assurance of SIB-IMRT with IGRT treatment and verifying appropriateness of PTV margins in current use at our institution or in other institutions using Helical TomoTherapy with organ immobilization procedures.

For prostate cancer patients who require radiation to pelvic lymphatics as well as prostate, using whole pelvic SIB-IMRT with Helical TomoTherapy is a feasible and effective modality especially while limiting intrapelvic organ motion by bladder emptying, endorectal ballooning, and daily enema. In the present study, there were no correlated factor for geometric shifts and geometrical shifts and degree of shifts were unchanged over fractions. Further studies on larger populations, with longer follow-up time, are needed to verify the actual treatment outcomes and contributing factors.
